# Comparative therapeutic efficacy of interferon alfa-2b and combination lopinavir/ritonavir plus interferon alfa-2b against SARS-CoV-2

**DOI:** 10.1186/s12879-021-06595-6

**Published:** 2021-08-30

**Authors:** Jingyuan Liu, Chunjing Du, Lin Pu, Pan Xiang, Haofeng Xiong, Wen Xie, Zhihai Chen, Ang Li

**Affiliations:** 1 Department of Critical Care Medicine, Beijing Ditan Hospital, Capital Medical University, No. 8 Jingshundong Street, Chaoyang District, Beijing, 100015 People’s Republic of China; 2 Center of Liver Diseases, Beijing Ditan Hospital, Capital Medical University, Beijing, People’s Republic of China; 3 Center of Infectious Disease, Beijing Ditan Hospital, Capital Medical University, Beijing, People’s Republic of China

**Keywords:** Coronavirus disease 2019 (COVID-19), Severe acute respiratory syndrome coronavirus 2 (SARS-CoV-2), Virus, Pneumonia

## Abstract

**Background:**

The outbreak of coronavirus disease 2019 (COVID-19) posed an enormous threat to public health. The use of antiviral drugs in patients with this disease have triggered people’s attentions. Whether interferon alfa-2b or lopinavir/ritonavir (LPV/r) plus interferon alfa-2b treatment can against SARS-CoV-2 was unknown. The objectives of this study was to evaluate the efficacy and safety of interferon alfa-2b and LPV/r plus interferon alfa-2b for SARS-CoV-2 infection in adult patients hospitalized with COVID-19.

**Methods:**

This is a retrospective cohort study of 123 patients confirmed SARS-CoV-2 infection by PCR on nasopharyngeal swab and symptoms between Jan. 13 and Apr. 23, 2020. All patients received standard supportive care and regular clinical monitoring. Patients were assigned to standard care group (n = 12), interferon alfa-2b group (n = 44), and combination LPV/r plus interferon alfa-2b group (n = 67). The primary endpoints were duration of required oxygen support and virus clearance time. Associations between therapies and these outcomes were assessed by Cox proportional hazards regression.

**Results:**

Baseline clinical characteristics were not significantly different among the three groups (*P* > 0.05). No significant associations were observed between LPV/r/interferon alfa-2b and faster SARS-CoV-2 RNA clearance (HR, 0.85 [95% confidence interval (CI) 0.45–1.61]; *P* = 0.61 in interferon alfa-2b group vs HR, 0.59 [95% CI 0.32–1.11]; *P* = 0.10 in LPV/r plus interferon alfa-2b group). Individual therapy groups also showed no significant association with duration of required oxygen support. There were no significant differences among the three groups in the incidence of adverse events (*P* > 0.05).

**Conclusions:**

In patients with confirmed SARS-CoV-2 infection, no benefit was observed from interferon alfa-2b or LPV/r plus interferon alfa-2b treatment. The findings may provide references for treatment guidelines of patients with SARS-CoV-2 infection.

## Background

COVID-19, caused by severe acute respiratory syndrome coronavirus 2 (SARS-CoV-2), poses an enormous threat to public health [1–4]. Since the first case of SARS-CoV-2 was published in December 2019, the number of laboratory-confirmed cases are escalating daily, with rampant spread of the virus to more than 200 countries and territories [5, 6]. COVID-19 cases are frequently associated with respiratory and multiorgan dysfunction that can result in death [7–9]. Accordingly, the use of antiviral drugs in patients with this disease has raised numerous questions and critical considerations, especially concerning whether currently available antiviral drugs can be used to effectively cure this disease.

Results of previous research showed that the protease inhibitor lopinavir/ritonavir (LPV/r), in combination with interferon, exhibited modest activity against both SARS-CoV and Middle East respiratory syndrome (MERS)-CoV [10–12]. However, a recent study by Cao and colleagues suggested that LPV/r alone contributed a limited, but clearly therapeutic benefits against COVID-19 [13]. Considering that coronaviruses can hijack the antiviral responses of type I interferon through structural and non-structural proteins, the use of interferon could potentially provide an effective treatment strategy to target and eliminate SARS-CoV-2 [14, 15]. However, Channappavanar et al. have demonstrated that delayed interferon-I expression was detrimental in the context of SARS-CoV-1 infection in mice [8, 16]. In addition, retrospective studies of interferon combined with ribavirin failed to show any obvious benefit in patients with MERS [12, 17]. Taken together, these findings emphasize that LPV/r and interferon remain controversial for treatment of COVID-19, and it is therefore imperative to fully investigate their therapeutic potential against this disease.

The purpose of this study was to evaluate the efficacy and safety of interferon alfa-2b and LPV/r plus interferon alfa-2b for SARS-CoV-2 infection in adult patients hospitalized with COVID-19.

## Methods

### Study design and participants

This retrospective cohort study was conducted on subjects with confirmed SARS-CoV-2 infection admitted to Beijing Ditan Hospital from January 13 to April 23, 2020. The institutional review boards approved this study and patient-level informed consent was waived due to its retrospective nature. SARS-CoV-2 infection was diagnosed by RT-PCR assays of respiratory tract samples from nasopharyngeal swabs performed by the local Center for Disease Control or by our institutional laboratory. The results were considered positive when the cycle threshold (Ct) values of open reading frame 1ab (ORF1ab) and nucleocapsid protein (N) gene exceeded 38. Pneumonia was defined as new, lower respiratory tract symptoms such as fever or chills, cough or shortness of breath, and new focal chest signs, coinciding with onset or progressive pulmonary infiltrates in chest radiography. The severity of COVID-19 was defined in accordance with the China’s COVID-19 management guidelines (version 7.0).

**Fig. 1:**
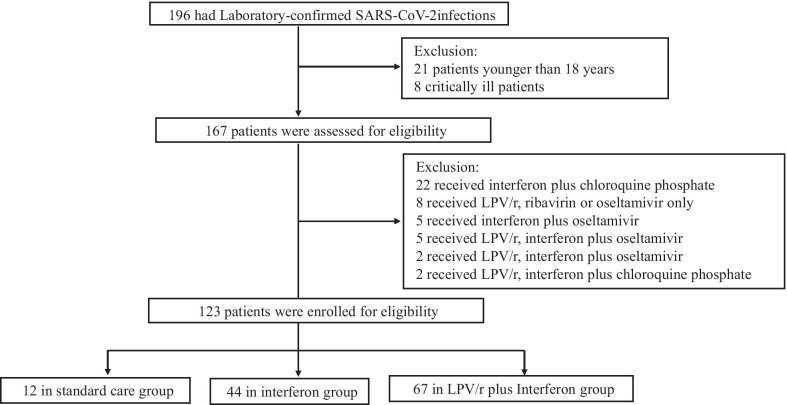
Flowchart of study population and exclusion criteria

All confirmed patients were offered treatment with standard care including, as necessary, supplemental oxygen, antibiotic agents, or traditional Chinese medicine. The potential antiviral therapies for SARS-CoV-2 were assigned in two groups: patients receiving LPV/r (500 mg twice daily, orally) plus interferon alfa-2b (interferon, 5 million units twice daily, nebulization) or patients receiving interferon alfa-2b, alone. Initially, a total of 196 cases were identified (Fig. 1). Among these, cases were excluded (n = 29) for patients who were younger than 18 years and critically ill patients. Among the 167 remaining participants, cases were excluded (n = 44) on the basis of treatment with other antiviral therapy including oseltamivir, chloroquine phosphate, and/or ribavirin. Subsequently, 123 participants were enrolled in the study. The two main exposure groups were divided as follows: LPV/r plus interferon alfa-2b therapy (n = 67), defined as the combined use of LPV/r and interferon alfa-2b, and interferon alfa-2b alone (n = 44). The comparator group received treatment with standard care without the use of LPV/r and interferon alfa-2b (n = 12). The final groups for the 123 included cases were: standard care group (n = 12), interferon alfa-2b group (n = 44), LPV/r plus interferon alfa-2b group (n = 67).

### Data collection

Data on patients’ demographics, underlying comorbidities, clinical presentation, oxygen-support requirements, and laboratory results were recorded. The time to SARS-CoV-2 RNA clearance in nasopharyngeal swabs of patients were also assessed. Nasopharyngeal swabs should be performed at least once every 3–5 days, depending on the patient’s clinical presentation. When patient’s symptoms were obviously relieved, a nasopharyngeal swab would be performed at an interval of 1–2 days. Virus clearance was defined as the time from admission until the RT-PCR assay returned negative results in two successive tests. To assess the safety profile of interferon alfa-2b and LPV/r plus interferon alfa-2b in COVID-19 patients, the incidence of nausea, diarrhea, rash and white blood cell counts, neutrophil counts, hemoglobin, platelet counts, alanine aminotransferase, bilirubin, and creatinine kinase were recorded. The LPV/r and interferon alfa-2b safety profile was assessed by evaluating the occurrence of nausea, diarrhea, rash, serial white blood cell count, neutrophil count, hemoglobin, platelet count, aminotransferase, bilirubin, and creatinine kinase according to the National Cancer Institute Common Terminology Criteria for Adverse Events, version 5.0.

### Outcomes

The primary endpoint for this study was the duration of oxygen-support requirement and virus clearance time.

### Statistical analysis

All analyses were conducted with IBM SPSS Statistics, version 19.0 (SPSS Institute, Chicago IL, USA). Analyses of categorical variables were conducted by the Chi-square (χ^2^) and Fisher’s exact tests. Normally distributed variables were compared using the Student’s t-test, whereas non-normally distributed variables were analyzed by the Kruskal–Wallis test. Two-sided P values of 0.05 or less were considered statistically significant. The times to oxygen-support requirement and virus clearance were visualized by Kaplan–Meier plot. Associations between therapies and these outcomes were assessed by Cox proportional hazards regression.

## Results

### Baseline characteristics of the patients

The baseline demographic and clinical characteristics of the 123 patients with SARS-CoV-2 are shown in Table 1. A total of 62 patients (50.41%) were men, with an age range of 18 to 92 years, and a median age of 41 years (interquartile range, 32 to 57). The number of comorbidities was 55 (44.72%), and hypertension accounted for the majority. At baseline, the majority of subjects were classified as mild patients (105 [85.37%]), with relatively fewer severe patients (18 [14.63%]). Among the patients, 82.93% presented with fever, although the median body temperature on admission was 37.0 °C (interquartile range, 36.5 °C to 37.6 °C). The median time from symptoms onset to hospitalization and treatment initiation were both 5 days (interquartile range, 2 to 7 days and 3 to 8 days, respectively). The duration of treatment in interferon group and LPV/r plus interferon group were 7 days and 11 days respectively (*P* = 0.054). A total of 54 patients (43.90%) enrolled requiring oxygen-support, with low-flow supplemental oxygen accounting for a majority of these patients (39.84%). In terms of laboratory results, blood indices of COVID-19 patients were within the normal range on the admission, including peripheral white cell count, platelets, C-reactive protein, serum creatinine, aspartate transaminase, alanine transaminase, bilirubin, LDH, and creatine kinase. Patients in the three groups were not significantly different in age, sex ratio, comorbidities, and baseline laboratory results at enrollment (*P* > 0.05).

**Table 1:** Baseline characteristics of 123 patients with SARS-Cov-2 infection

	Standard care (n = 12)	Interferon (n = 44)	LPV/r plus interferon (n = 67)	Total patients (n = 123)	P-value
Male	4 (33.33)	23 (52.27)	35 (52.24)	62 (50.41)	0.46
Age (years)	59.50 (37, 66.25)	42.50 (30.75, 58.50)	40.00 (32.00, 51.00)	41.00 (32.00, 57.00)	0.05
Comorbidities	6 (50)	22 (50)	27 (40.30)	55 (44.72)	0.56
Diabetes	0	2 (4.55)	2 (2.99)	4 (3.25)	
Hypertension	2 (16.67)	7 (15.91)	10 (14.93)	19 (15.45)	
Cardiovascular disease	0	1 (2.27)	1 (1.49)	2 (1.63)	
Hyperlipemia	1 (8.33)	1 (2.27)	1 (1.49)	3 (2.44)	
Cerebrovascular disease	0	2 (4.55)	3 (4.48)	5 (4.07)	
Previous surgery	1 (8.33)	5 (11.36)	7 (10.45)	13 (10.6)	
Other disease	2 (16.67)	4 (9.09)	3 (4.48)	9 (7.32)	
Fever	9 (75)	35 (79.50)	58 (86.57)	102 (82.93)	0.47
Body temperature (°C)	37.10 (36.50, 37.95)	36.80 (36.60, 37.25)	37.20 (36.50, 37.70)	37.00 (36.50, 37.60)	0.34
Severity category at admission					0.15
Mild	8 (66.67)	38 (86.36)	59 (88.06)	105 (85.37)	
Severe	4 (33.33)	6 (13.64)	8 (11.94)	18 (14.63)	
Time from symptoms onset to hospitalization	4 (2, 8)	5 (2, 7)	5 (2, 8)	5 (2, 7)	0.98
Time from symptoms onset to treatment		6 (3, 10)	5 (3, 8)	5 (3, 8)	0.86
Duration of treatment		7 (3, 19)	11 (6, 18)	10 (5, 18)	0.05
Oxygen therapy	6 (50)	22 (50)	26 (38.81)	54 (43.90)	0.46
Low flow oxygen	4 (33.33)	20 (45.45)	25 (37.31)	49 (39.84)	
High flow oxygen	2 (16.67)	2 (4.55)	1 (1.49)	5 (4.07)	
White-cell count (× 10^9^/L)	4.49 (3.31, 6.09)	4.39 (3.50, 5.66)	4.89 (4.07, 5.84)	4.66 (3.75, 5.76)	0.40
Neutrophil count (× 10^9^/L)	2.84 (2.07, 4.66)	2.75 (2.04, 3.79)	3.26 (2.30, 4.14)	3.05 (2.17, 3.85)	0.67
Lymphocyte count (× 10^9^/L)	1.19 (0.83, 1.44)	1.17 (0.85, 1.37)	1.19 (0.95, 1.72)	1.19 (0.93, 1.53)	0.47
Monocyte count (× 10^9^/L)	0.30 (0.21, 0.43)	0.30 (0.22, 0.41)	0.36 (0.25, 0.43)	0.33 (0.24, 0.42)	0.36
Platelet count (× 10^9^/L)	181.50 (170.50, 239.75)	174.00 (134.00, 250.00)	203.00 (151.00, 239.00)	190.00 (150.00, 244.00)	0.60
CRP (mg/L)	13.65 (1.15, 36.88)	8.05 (1.10, 26.80)	11.40 (1.40, 31.90)	10.20 (1.20, 31.40)	0.90
Serum creatinine (μmol/L)	56.15 (48.30, 79.93)	68.70 (57.48, 73.75)	68.50 (56.30, 79.70)	68.50 (55.80,78.50)	0.28
Aspartate aminotransferase (U/L)	23.00 (11.90, 32.50)	24.30 (18.40, 34.00)	24.50 (17.08, 32.00)	24.30 (17.83, 32.13)	0.75
Alanine aminotransferase (U/L)	27.90 (17.95, 37.48)	23.10 (14.90, 34.70)	24.00 (17.60, 32.20)	24.00 (17.10, 32.78)	0.79
TBIL (U/L)	9.40 (6.90, 14.00)	10.60 (7.90, 13.70)	10.00 (8.05, 14.70)	10.40 (7.80, 14.00)	0.56
LDH (U/L)	214.40 (193.30, 314.70)	202.55 (179.90, 266.50)	220.30 (185.70, 301.40)	214.35 (183.55, 297.58)	0.49
Creatine kinase (U/L)	59.90 (32.23, 120.00)	67.80 (42.33, 121.13)	78.20 (57.60, 132.80)	70.00 (50.00, 132.20)	0.13

Data are median (IQR), n (%)

*CRP* C-reactive protein, *LDH* lactate dehydrogenase, *TBIL* total bilirubin

### Treatment outcomes

**Fig. 2:**
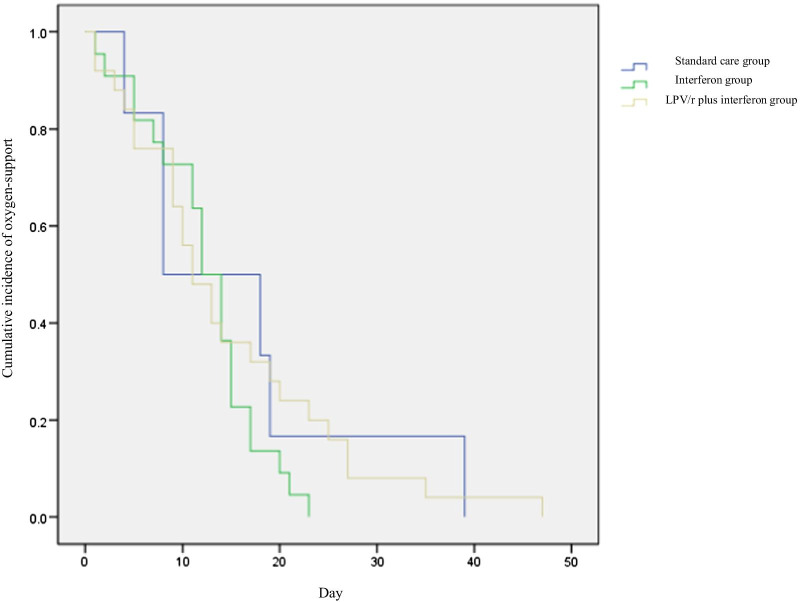
Cumulative incidence of required oxygen support from baseline

**Fig. 3:**
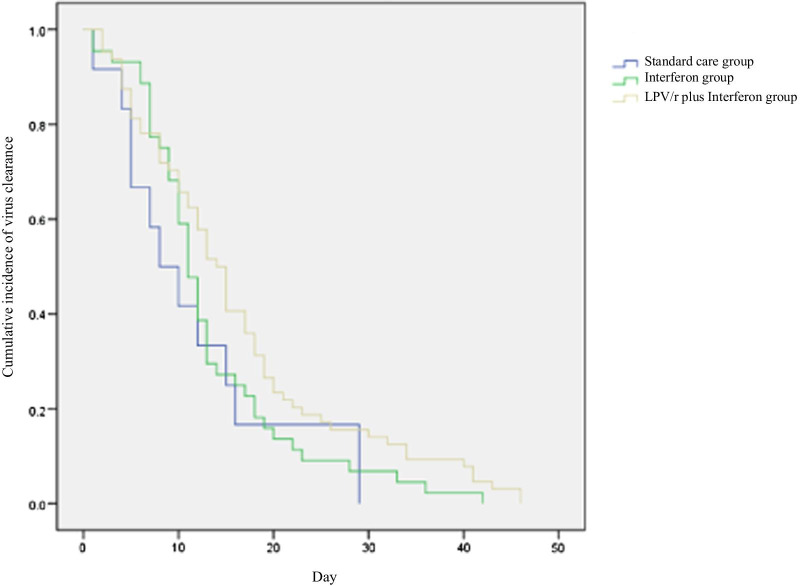
Cumulative incidence of virus clearance from baseline

Crude data analysis showed no significant differences in duration of required oxygen support between therapy groups and standard care group (median, 13 days [interquartile range: 8, 16] in interferon alfa-2b group; 11 days [interquartile range: 7, 22] in LPV/r plus interferon alfa-2b group vs 13 days [interquartile range: 7, 24 in standard care group]; *P* = 0.98; Table 2). In comparisons of time to virus clearance, no significant differences were observed among the three groups. Using Cox proportional hazards regression adjusted for baseline covariate, no significant association was found between LPV/r /interferon alfa-2b and faster SARS-CoV-2 RNA clearance (HR, 0.85 [95% CI 0.45–1.61]; *P* = 0.61 in interferon alfa-2b group vs HR, 0.59 [95% CI 0.32–1.11]; *P* = 0.10 in LPV/r plus interferon alfa-2b group, Table 3, Fig. 2). The duration of required oxygen support similarly showed no significant association between either therapy group and standard care group (HR, 1.46 [95% CI 0.57–3.75]; *P* = 0.43 in interferon alfa-2b group vs HR, 1.06 [95% CI 0.43–2.63]; *P* = 0.90 in LPV/r plus interferon alfa-2b group, Table 3, Fig. 3).

**Table 2:** The comparison between treatment groups and standard group in the duration of oxygen-support and CoV RNA clearance time

	Standard care (n = 12)	Interferon (n = 44)	LPV/r plus interferon (n = 67)	Total patients (n = 123)	P-value
Oxygen-support duration	13 (7, 24)	13 (8, 16)	11 (7, 22)	12 (8, 19)	0.98
Viral clearance	9 (5, 15)	11 (8, 17)	14 (8, 20)	12 (7, 18)	0.33

Data are median (IQR)

**Table 3:** Correlation analysis of treatment groups with the duration of oxygen-support and CoV RNA clearance

Variable	Oxygen-support duration		Viral clearance	
	HR (95% CI)	P-value	HR (95% CI)	P-value
Interferon/standard care	1.46 (0.57, 3.75)	0.43	0.85 (0.45, 1.61)	0.61
LPV/r plus Interferon/standard care	1.06 (0.43, 2.63)	0.90	0.59 (0.32, 1.11)	0.10
Interferon/ LPV/r plus interferon	1.38 (0.75, 2.54)	0.30	1.43 (0.96, 2.12)	0.08

*CI* confidence interval

**Fig. 4:**
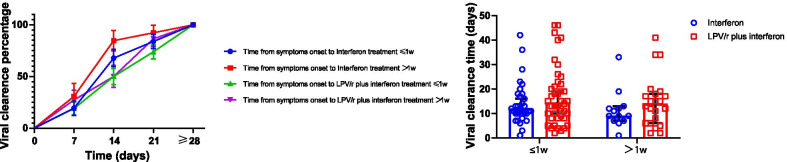
Viral-clearance of therapy groups from symptoms onset to treatment initiation. **A** Viral-clearance rates of therapy groups from symptoms onset to treatment initiation. **B** Viral-clearance time of therapy groups within/after the 1st week from symptoms onset to treatment initiation

The differences in virus clearance of therapy groups from symptoms onset to treatment initiation within/after the 1st week from symptoms onset to start of treatment were further analyzed (Fig. 4a, b). Indeed, there was no significant differences in virus clearance percentage between interferon group (19.36%, CI [16.78, 28.82]) and LPV/r plus interferon alfa-2b group (19.05%, CI [15.43, 24.64]) within the 1st week from symptoms onset to start of treatment (*P* > 0.05). Meanwhile, no differences were found in viral clearance time between interferon group and LPV/r plus interferon alfa-2b group in time from symptoms onset to treatment within 1w (12 and 14 respectively; *P* > 0.05) and after 1w (9 and 14 respectively; *P* > 0.05).

### Safety endpoints

LPV/r and interferon alfa-2b therapy was well-tolerated by the exposed group, with no premature discontinuation due to adverse effects. There were no significant differences among the three groups in the incidence of nausea, diarrhea, rash, leukopenia, neutropenia, anemia, thrombocytopenia, increased creatinine, or other adverse events (Table 4). However, the incidence of elevated creatine kinase was higher in the exposed groups over the course of the therapy (15.91% in interferon alfa-2b group vs 20.90% in LPV/r/ interferon alfa-2b group) than in the standard care group (0), although this difference was not statistically significant. Aside from anemia and increased bilirubin, the occurrence of adverse effects was lower in the standard care group than in the therapy groups.

**Table 4:** Summary of adverse events in the enrolled patients

	Standard care (n = 12)	Interferon (n = 44)	LPV/r plus interferon (n = 67)	Total patients (n = 123)	P-value
Any event					
Nausea	0	1 (2.27)	5 (7.46)	6 (4.88)	0.33
Diarrhea	0	4 (9.09)	11 (16.42)	15 (12.20)	0.20
Rash	0	2 (4.55)	2 (2.99)	4 (3.25)	0.72
Leukopenia	0	8 (18.18)	8 (11.94)	16 (13.01)	0.23
Neutropenia	0	6 (13.64)	6 (8.96)	12 (9.76)	0.35
Anemia	1 (8.33)	1 (2.27)	3 (4.48)	6 (4.88)	0.12
Thrombocytopenia	0	2 (4.55)	1 (1.49)	6 (4.88)	0.25
Increased creatinine	0	2 (4.55)	1 (1.49)	3 (2.44)	0.50
Increased aminotransferase	0	2 (4.55)	1 (1.49)	3 (2.44)	0.50
Increased bilirubin	1 (8.33)	2 (4.55)	9 (13.43)	12 (9.76)	0.30
Increased creatine kinase	0	7 (15.91)	14 (20.90)	21 (17.07)	0.20

Data are n (%)

## Discussion

Effective interventions for treating patients with SARS-CoV-2 infection are still urgently needed. While the benefits of LPV/r and interferon alfa-2b were suggested by preclinical studies, the present study showed that neither interferon alfa-2b nor LPV/r plus interferon alfa-2b in addition to standard care were associated with duration of required oxygen support or virus clearance time compared with standard care alone. Thus, it remains unclear as to whether interferon alfa-2b or LPV/r plus interferon alfa-2b treatment can provide clinical benefits against COVID-19 given the promising initial results. In light of the urgent need for COVID-19 treatments, further study of LPV/r and interferon alfa-2b is still warranted based on its FDA approval and safety profile as an antiviral.

Prior research from Alhazzani et al. showed that LPV/r /interferon therapy was not associated with clinical improvement or CoV RNA clearance, which was consistent with the results of our study [13, 17, 18]. Moreover, the Society of Critical Care Medicine issued recommendations against the use of LPV/r in critically ill COVID-19 patients [19]. However, previous studies also showed that LPV/r and interferon alfa-2b lead to clinical improvement for patients with SARS-CoV or MERS-CoV infection [10–12, 20], although the reasons why similar treatments lead to different clinical outcomes and efficacy in viral RNA clearance are uncertain. The non-randomized design, differences in baseline characteristics, and small sample size are potentially related to the observed inconsistencies. Additionally, owing to the non-standard initiation of therapy, such studies are prone to two biases: indication bias and immortal time bias.

Although no significant differences were observed among the three groups over the hospitalization stay in these adverse events, notably, we found that the incidence of increased creatine kinase was higher in patients treated with interferon alfa-2b or LPV/r plus interferon alfa-2b than in the standard care group. This difference between treatment and comparator groups may be related to interferon alfa-2b therapy, which is consistent with previous reports [21, 22]. Contradicting this conclusion, recent research by Pan et al. proposed that the elevation in creatine kinase was correlated with viral infection [23]. Further in vitro or animal model studies may confirm or exclude interferon alfa-2b as the cause of elevated creatinine kinase in COVID-19 patients.

The limitations of this study are that it is retrospective and non-randomized. Inevitably, selection and unmeasured confounding bias could not be completely excluded, and further interventions should be ideally evaluated in randomized, controlled clinical trials. However, in the context of emerging disease and epidemic, it is generally accepted that such methods are not always practical. In addition, the small sample size of the control group is also a limitation of this study. Thus, further research should use a larger sample size for the control group to strengthen the accuracy of the results.

## Conclusion

In summary, in patients with confirmed SARS-CoV-2 infection, no clinical benefit was observed from treatment with interferon alfa-2b or LPV/r plus interferon alfa-2b. The findings may provide references for treatment guidelines of patients with SARS-CoV-2 infection.

## Data Availability

The datasets used and/or analysed during the current study are available from the corresponding author on reasonable request.

## Abbreviations

COVID-19: Coronavirus disease 2019
CRP: C-reactive protein
LDH: Lactate dehydrogenase
LPV/r: Lopinavir/ritonavir
MERS: Middle East respiratory syndrome
SARS-CoV-2: Severe acute respiratory syndrome coronavirus 2
TBIL: Total bilirubin
WBC: White blood cell
